# Quinone binding sites of cyt *bc* complexes analysed by X-ray crystallography and cryogenic electron microscopy

**DOI:** 10.1042/BST20190963

**Published:** 2022-03-31

**Authors:** Wei-Chun Kao, Carola Hunte

**Affiliations:** 1 Institute of Biochemistry and Molecular Biology, ZBMZ, Faculty of Medicine, University of Freiburg, Freiburg, Germany; 2 Signalling Research Centres BIOSS and CIBSS, University of Freiburg, Freiburg, Germany

**Keywords:** cryo electron microscopy, crystallography, cytochrome *bc* complex, electron transport chain, menaquinone, ubiquinone

## Abstract

Cytochrome (cyt) *bc*_1_, *bcc* and *b*_6_*f* complexes, collectively referred to as cyt *bc* complexes, are homologous isoprenoid quinol oxidising enzymes present in diverse phylogenetic lineages. Cyt *bc*_1_ and *bcc* complexes are constituents of the electron transport chain (ETC) of cellular respiration, and cyt *b*_6_*f* complex is a component of the photosynthetic ETC. Cyt *bc* complexes share in general the same Mitchellian Q cycle mechanism, with which they accomplish proton translocation and thus contribute to the generation of proton motive force which drives ATP synthesis. They therefore require a quinol oxidation (Q_o_) and a quinone reduction (Q_i_) site. Yet, cyt *bc* complexes evolved to adapt to specific electrochemical properties of different quinone species and exhibit structural diversity. This review summarises structural information on native quinones and quinone-like inhibitors bound in cyt *bc* complexes resolved by X-ray crystallography and cryo-EM structures. Although the Q_i_ site architecture of cyt *bc*_1_ complex and cyt *bcc* complex differs considerably, quinone molecules were resolved at the respective Q_i_ sites in very similar distance to haem *b*_H_. In contrast, more diverse positions of native quinone molecules were resolved at Q_o_ sites, suggesting multiple quinone binding positions or captured snapshots of trajectories toward the catalytic site. A wide spectrum of inhibitors resolved at Q_o_ or Q_i_ site covers fungicides, antimalarial and antituberculosis medications and drug candidates. The impact of these structures for characterising the Q cycle mechanism, as well as their relevance for the development of medications and agrochemicals are discussed.

## Introduction

**Figure 1.**
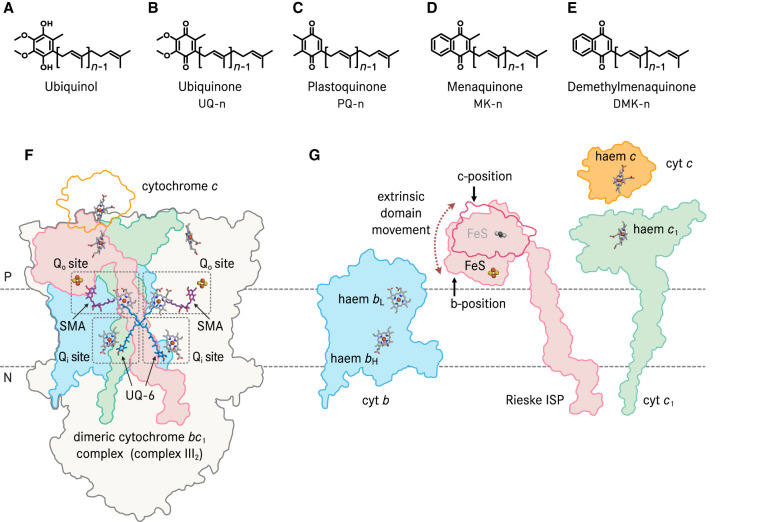
Cytochrome *bc*_1_ complex and its substrates. Chemical structures of (**A**) ubiquinol, (**B**) ubiquinone, (**C**) plastoquinone, (**D**) menaquinone and (**E**) demethylmenaquinone. The number of isoprenoid units is denoted as *n*. Ubiquinol is the reduced form of ubiquinone. (**F**) Cytochrome (cyt) *bc*_1_ complex from *Saccharomyces cerevisiae* and its relative position in the inner mitochondrial membrane. The location of the quinol oxidation Q_o_ site is marked by the inhibitor stigmatellin (SMA) which was co-crystallised with cyt *bc*_1_ complex (pdb 2ibz). The location of the quinone reduction Q_i_ site is indicated by ubiquinone-6 (UQ-6) which was co-isolated with the enzyme (pdb 2ibz). Soluble cyt *c* is a substrate of cyt *bc*_1_ complex. Its docking position is illustrated based on the X-ray structure of the electron-transfer complex (pdb 3cx5). The three membrane-bound catalytic subunits of one protomer of the dimeric enzyme, namely cyt *b*, Rieske iron-sulfur protein (ISP) and cyt *c*_1_ as well as the substrate cyt *c*, are separately illustrated in (**G**) The extrinsic domain of Rieske ISP undergoes diffusional movement and its position close to cyt *b* (b-position, pdb 2ibz) and close to cyt *c*_1_ (c-position, pdb 1be3) are both indicated. The iron-sulfur cluster (FeS) is depicted in gray scale at the c-position and the extrinsic domain at the c-position is only outlined. P and N indicate the electropositive and -negative sides of the inner mitochondrial membrane. Iron atoms are depicted in brown, sulfur atoms are shown in yellow.

Isoprenoid quinones are a family of natural electron and proton carriers present in prokaryotic cellular membranes, in the mitochondrial inner membrane and in the chloroplast thylakoid membrane [1–3]. The various isoprenoid quinone species differ in their water-soluble ring system and the length of the hydrophobic isoprenoid tails [4–6] (Figure 1A–E). The electrochemically active part of this family of molecules is the quinone ring system, which accepts two electrons and two protons to become the fully reduced quinol (Figure 1A), while the highly hydrophobic isoprenoid tail enhances its solubility in biological membranes. Isoprenoid quinone and quinol are substrates of respiratory chain and photosynthetic enzymes [7,8].

In the electron transport chain (ETC) of cellular respiration, NADH dehydrogenase (complex I) and succinate dehydrogenase (complex II) reduce quinone, harnessing the energy of redox equivalents obtained from metabolism while cytochrome *bc*_1_ complex (cyt *bc*_1_ complex, complex III) oxidises quinol and transfer the electrons to cytochrome *c* oxidase (cyt *c* oxidase, complex IV) via the electron carrier protein cytochrome (cyt) *c*. The cyt *c* oxidase catalyses the reduction in dioxygen to water. NADH dehydrogenase and cyt *bc*_1_ complex couple quinone redox chemistry to proton translocation across the inner mitochondrial or bacterial cellular membrane to generate an electrochemical proton gradient and thereby power ATP synthesis [1,3]. In the ETC of photosynthesis, photosystem II utilises light energy to reduce quinone, and cyt *b*_6_*f* complex [9–11], a homologue of cyt *bc*_1_ complex, oxidises quinol and passes electrons to photosystem I. Photosystem II and cyt *b*_6_*f*complex create a proton gradient across the chloroplast thylakoid membrane or the cyanobacterial plasma cellular membrane for ATP synthesis [12]. Therefore, cyt *bc*_1_ and cyt *b*_6_*f* complex are substantial contributors to the driving forces of cellular energy conversion.

Cyt *bc*_1_ and cyt *b*_6_*f*complexes form a large group of enzymes which all include a Rieske iron-sulfur protein (ISP), a *b*-type cytochrome (cyt *b or* cyt *b*_6_-SUIV, ‘subunit four') and a *c*-type cytochrome (cyt *c*_1_, cyt *f* or di-haem cyt *cc*) as the core catalytic module (Figure 1F,G) [2,13,14]. Cyt *bc*_1_ and cyt *b*_6_*f* complexes are found in organisms from diverse phylogenetic clades [13], and they differ in composition in respect to number and types of peripheral subunits [10,15–17]. In actinobacteria, the catalytic Rieske ISP, cyt *b*, cyt *cc* and the cyt *aa*_3_ oxidase plus peripheral subunits comprise the cyt *bcc*-*aa*_3_ supercomplex [18,19]. Therefore, they are collectively referred to as cyt *bc* complexes in this mini-review.

**Figure 2.**
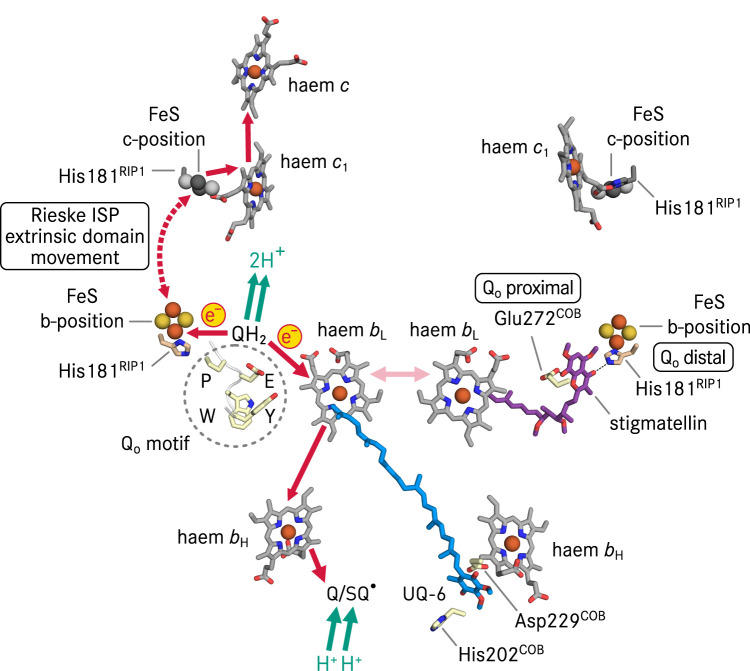
The Q cycle mechanism. Catalytic centres of dimeric cyt *bc*_1_ complex from *S. cerevisiae* are illustrated in two ways. The structure of the right half shows the inhibitor stigmatellin and the natural substrate UQ-6 in the X-ray structure (pdb 2ibz) as well as their hydrogen bonding partners. His181^RIP1^ is also a ligand of the iron-sulfur cluster (FeS). Stigmatellin represents the position of a transition state of ubiquinol (QH_2_) oxidation in the Q_o_ site, and UQ-6 indicates the position of ubiquinone/semiubiquinone (Q/SQ^•^) in the Q_i_ site. The structure of the left schematically shows the Q cycle mechanism. The four highly conserved residues of cyt *b* (COB): Pro271^COB^ (P), Glu272^COB^ (E), Trp273^COB^ (W) and Tyr274^COB^ (Y) in the Q_o_ site form the Q_o_ motif [14]. Electron transfer in cyt *bc*_1_ complex can also cross the dimeric enzyme (pink arrow) [111]. Owing to the large-scale movement of the extrinsic domain (ED) of Rieske iron-sulfur protein (RIP1), the FeS is shown at two positions, the b-position (based on pdb 2ibz) close to the Q_o_ site quinol and the c-position close to haem *c*_1_ (based on pdb 1be3). Whereas the Q_o_ site ubiquinol releases two electrons and two protons upon oxidation, only one electron is transferred to Q_i_ site, therefore the full reduction in the Q_i_ site quinone requires oxidation of a second ubiquinol molecule at the Q_o_ site and the uptake of two protons. The exact sequence of protonation steps at the Q_i_ site is not differentiated in this simplified scheme. Iron atoms are depicted in brown, sulfur atoms are shown in yellow. The FeS and its ligand His181^RIP1^ at the c-position are shown in gray scale. Hydrogen bonds are depicted as dashed lines. Electron transfer pathways are shown in red, and proton release and uptake routes are in green.

In respiratory and photosynthetic ETCs, the overall forward reaction of cyt *bc* complexes is to oxidise quinol molecules and to reduce cytochrome *c* or plastocyanin, which will further transfer the electron to cyt *c* oxidase or photosystem I, respectively. Cyt *bc* complexes do not directly pump protons across the membrane such as for instance cyt *c* oxidases, instead, proton translocation is achieved through the Mitchellian Q cycle mechanism (Figure 2) [2,11,20–24]. As the first step in a Q cycle, a quinol molecule is oxidised at the quinol oxidation (Q_o_) site of cyt *bc* complex close to the positive side (P-side) of the membrane (Figure 1F). Next, using the mitochondrial cyt *bc*_1_ complex as an example, one electron of ubiquinol is transferred to the Rieske iron-sulfur cluster (FeS) and subsequently to haem *c*_1_. The extrinsic domain of Rieske ISP undergoes a substantial conformational change [16,25–27] to bridge the 24 Å distance between the Q_o_ site quinol and haem *c*_1_ (Figure 2). Physiological electron transfer rates typically require a maximal distance of 14 Å between electron donor and acceptor [28,29]. The other electron is routed through the low potential haem *b*_L_, the high potential haem *b*_H_ and reduces a quinone molecule in the Q_i_ site to a semiquinone radical (SQ^•^). In this process, the Q_o_ site quinol releases two protons to the P-side of the membrane and the complete reduction and protonation of a quinone molecule in the Q_i_ site needs oxidation of a second quinol at the Q_o_ site and proton uptake from the N-side of the membrane. Consequently, bifurcated electron transfer must be achieved upon quinol oxidation to enable the Q cycle, i.e. the highly reactive SQ^•^ at the Q_o_ site must be controlled to avoid short circuits [2,29–34] which lead to futile bypass reactions which would lower the efficiency of cellular respiration and can generate reactive oxygen species [29] that can cause oxidative damage to the cell [35].

Experimental structures of cyt *bc* complexes are essential to understand the molecular basis for efficient and safe electron and proton transfer mechanisms at Q_o_ and Q_i_ site. Position, geometry and distance of electron donors and acceptors, of substrate and analogous molecules as well as of prosthetic groups, are important to define electron transfer pathways [28]. Resolved positions of protonable amino acid side chains, hydronium ions (H_3_O^+^) or water molecules enable to identify proton transfer pathways [36]. Owing to the central role of cyt *bc* complexes in cellular respiration and in photosynthesis, structural biology studies of these complexes based on X-ray crystallography and cryogenic electron microscopy (cryo-EM) have delivered, over the years, a great number of experimental structures of mitochondrial cyt *bc*_1_ complexes [16,17,37–40] as well as of respiratory supercomplexes [41–48], alpha-proteobacterial cyt *bc*_1_ complexes [27,49,50], cyanobacterial [9] and chloroplast [10,51] cyt *b*_6_*f* complexes and actinobacterial cyt *bcc-aa*_3_ supercomplexes [19,52–54]. One should note that, the electrochemical properties of the redox-active centres of cyt *bc* complexes co-evolved with those of their native quinone substrates [13,55–57]. Hence, comparison of structures of cyt *bc* complexes with bound substrates sampled from a wide spectrum of organisms sheds light on the conserved structural basis of the Q cycle's quinone catalysis as well as on adaptations reflecting its molecular evolution, and may support development of medications precisely targeting different pathogens.

## Quinone binding positions at the Q_o_ site

In cyt *bc*_1_ and *b*_6_*f* complexes, the Q_o_ site is embedded in subunit cyt *b* and at the interface with the mobile extrinsic domain of Rieske ISP (Figs. 1F, 2). The native substrate at the Q_o_ site is quinol, the reduced form of quinone, and the oxidised reaction product quinone has to leave the catalytic position at the Q_o_ site. So far, native quinone or quinol molecules were not resolved at the catalytic Q_o_ site position in X-ray crystallography studies (Table 1), in particular because crystal formation requires a defined conformation of the complex, and the unrestrained motion of the extrinsic domain of Rieske ISP may hinder this process. Consequently, the characterisation of the binding mode of the substrate in the Q_o_ site was supported by the use of inhibitors, and three binding positions at the Q_o_ site were suggested [58]. The proximal position (Figure 2) was assigned with myxothiazol, which is hydrogen-bonded solely to Glu272 of cyt *b* (Glu272^COB^, yeast numbering) and shows no interaction to Rieske ISP [37]*.* The distal binding position (Figure 2) is exemplified by HHDBT, which is hydrogen-bonded to the iron-sulfur-cluster (FeS) ligand (His181^RIP1^) of the Rieske protein, and to Glu272^COB^ with a water-mediated hydrogen bond [59]. The third binding position is characterised with stigmatellin, which is hydrogen bonded directly to both Glu272^COB^ and His181^RIP1^ [17] (Figure 2). Stigmatellin also binds at the Q_o_ site of the cyt *bcc*-*aa*_3_ supercomplex of *Corynebacterium glutamicum* in a similar manner as in the mitochondrial cyt *bc*_1_ complexes [19], therefore it exhibits a conserved binding pose in the Q_o_ sites of cyt *bc* complexes which oxidise respectively ubiquinone or menaquinone. The Q_o_ site pocket is unlikely to accommodate two isoprenoid quinol molecules simultaneously due to spatial constraints, thus these aforementioned three inhibitor binding positions may reflect the locations of reaction intermediates in different oxidation or protonation states, as well as their interactions with potential proton acceptors [22,60,61]. One of the proton acceptors is His181^RIP1^, which undergoes a p*K*_a_ change dependent on the Rieske protein redox state [62,63]. The other hypothetical proton acceptor is Glu272^COB^*.* Its substitution with other residues by mutagenesis partially compromises the turnover of the enzyme [14] but its exact function remains elusive. Glu272^COB^ is the second residue of the Q_o_ motif of cyt *b*, a highly conserved motif of four consecutive amino acid residues (Figure 2) present in all cyt *bc* complexes with systematic phylogenetic variations (PEWY in mitochondrial cyt *b*) [14]. The type of residue at the second position of the Q_o_ motif is correlated with the redox midpoint potential of cyt *bc* complex cofactors as well as with the quinone species [14]. Substrate binding positions in experimental structures of cyt *bc* complexes from different organisms would be very important to derive the conserved structural basis of catalysis as well as species-specific adaptations.

**Table 1:** Structures of cyt *bc* complexes with bound native quinone molecule resolved

Position			Year	Complex type	pdb	Res (Å)	Method	Origin	Quinone
	Q_i_		1998	cyt *bc*_1_ complex	1bcc	3.16	X-ray	*Gallus gallus*	UQ-10
	Q_i_		2000	cyt *bc*_1_ complex	1ezv	2.30	X-ray	*Saccharomyces cerevisiae*	UQ-6
	Q_i_		2003	cyt *b*_6_*f* complex	1vf5	3.00	X-ray	*Mastigocladus laminosus*	PQ-9
	Q_i_		2005	cyt *bc*_1_ complex	1pp9	2.10	X-ray	*Bos taurus*	UQ-10
	Q_i_		2008	cyt *bc*_1_ complex	2qjy	2.40	X-ray	*Rhodobacter sphaeroides*	UQ-10
Q_o_	Q_i_		2018	Supercomplex III_2_/IV_2_	6adq	3.50	cryo-EM	*Mycobacterium smegmatis*	MK-9
Q_o_	Q_i_		2018	Supercomplex III_2_/IV_2_	6hwh	3.30	cryo-EM	*Mycobacterium smegmatis*	MK-9
Q_o_	Q_i_		2019	Supercomplex I/III_2_	6q9e	3.90	cryo-EM	*Ovis aries*	UQ-10
Q_o_	Q_i_		2019	cyt *b*_6_*f* complex	6rqf	3.60	cryo-EM	*Spinacia oleracea*	PQ-9
	Q_i_		2019	Supercomplex III_2_/IV	6giq	3.23	cryo-EM	*Saccharomyces cerevisiae*	UQ-6
	Q_i_		2019	Supercomplex III_2_/IV_2_	6hu9	3.35	cryo-EM	*Saccharomyces cerevisiae*	UQ-6
	Q_i_		2020	cyt *bc*_1_ complex	6kls	3.30	cryo-EM	*Aquifex aeolicus*	DMK-7
Q_o_	Q_i_		2021	cyt *bc*_1_ complex	7rja	3.00	cryo-EM	*Candida albicans*	UQ-10
Q_o_	Q_i_		2021	Supercomplex III_2_/IV_2_	7e1v	2.68	cryo-EM	*Mycobacterium tuberculosis/smegmatis*	MK-9
Q_o_	Q_i_	Q_c_	2021	Supercomplex III_2_/IV_2_	7q21	2.90	cryo-EM	*Corynebacterium glutamicum*	MK-9
	Q_i_	Q_c_	2022	Supercomplex III_2_/IV_2_	7qhm	2.80	cryo-EM	*Corynebacterium glutamicum*	MK-9
Q_o_	Q_i_	Q_c_	2022	Supercomplex III_2_/IV_2_	7qho	3.10	cryo-EM	*Corynebacterium glutamicum*	MK-9

Binding position was assigned according to authors’ descriptions in the original publications (see text for references). The pdb code, resolution (Res) and experimental method of each entry are sourced from RCSB PDB (https://www.rcsb.org).

**Figure 3.**
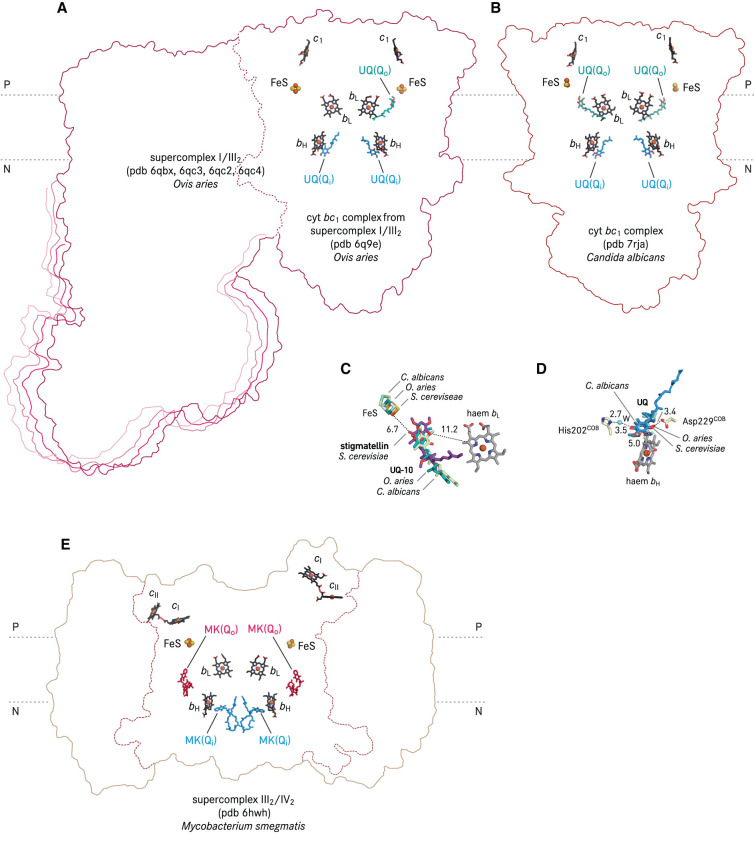
Positions of natural quinone molecules resolved in supercomplex I/III_2_, cyt *bc*_1_ complex and cyt *bcc*-*aa*_3_ supercomplex. (**A**) cryo-EM structure of cyt *bc*_1_ complex as part of the supercomplex I/III_2_ from sheep. The cryo-EM map of cyt *bc*_1_ complex was reconstructed from focused-refinement of four supercomplex I/III_2_ maps representing different conformational states [48], therefore the outlines of all four corresponding supercomplex structures were all illustrated. Co-ordinates of supercomplex I/III_2_ were superposed on one cyt *b* of pdb 6q9e using secondary structure maching in Coot [112]. (**B**) cryo-EM structure of cyt *bc*_1_ complex from *Candida albicans* [40]. (**C**) Comparison of stigmatellin and ubiquinone-10 (UQ-10) binding positions by superimposition of the co-ordinates of the cyt *bc*_1_ complex structure. Cyt *b* of the yeast (*S. cerevisiae*) cyt *bc*_1_ complex (pdb 2ibz, with stigmatellin [67]) was used as reference, and cyt *b* of pdb 6q9e (*O. aries*), and pdb 7rja (*C. albicans*) were superposed using secondary structure matching in Coot [112]. The FeS of all structures are shown, whereas only the haem *b*_L_ of pdb 2ibz is displayed for the sake of clarity. All distances are in Å. (**D**) Comparison of ubiquinone binding positions at the Q_i_ site. Water molecule is labelled as W. Haem *b*_H_ and the side chains of Asp229^COB^, His202^COB^ are from pdb 2ibz. (**E**) cryo-EM structure of cyt *bcc-aa*_3_ supercomplex (supercomplex III_2_/IV_2_) from *Mycobacterium smegmatis* (pdb 6hwh) [53]. For Figures 3–5, all the quinone types and locations are assigned according to the positions reported in the original publications. For cyt *bc* complexes resolved within a supercomplex, the boundary of the cyt *bc* complex is depicted in dashed lines, and only prosthetic groups of cyt *bc* complexes are shown. The electropositive and -negative sides of the membrane are indicated with P and N, respectively. Depending on the resolution and data quality, the isoprenoid units of quinones structurally resolved can deviate from the full length of native isoprenoid quinones of the given species.

Recently, native co-isolated quinone molecules at or in proximity to the Q_o_ site were identified in several cryo-EM structures of respiratory chain supercomplexes (Table 1). In a mammalian respiratory I/III_2_ supercomplex [48], which is composed of a NADH dehydrogenase (complex I) and a dimeric cyt *bc*_1_ complex (complex III_2_), an ubiquinone molecule was identified in the Q_o_ site which is distal to complex I, whereas the Q_o_ site proximal to the quinone reduction tunnel of complex I was unoccupied (Figure 3A). The authors proposed that the Q_o_ site close to complex I would accept ubiquinol reduced by complex I as they share the shortest diffusion distance [48]. The cryo-EM structure of cyt *bc*_1_ complex from *Candida albicans* contains a ubiquinone molecule in the Q_o_ site of both protomers [40] (Figure 3B). By superimposition of the mammalian supercomplex I/III_2_ with *Candida albicans* complex III, and yeast cyt *bc*_1_ complex co-crystallised with stigmatellin, a trajectory of Q_o_ site occupants can be deduced (Figure 3C). In comparison, stigmatellin reached deepest into the Q_o_ site pocket. The ubiquinone molecules resolved in the cryo-EM structures only partially overlap with the stigmatellin binding position. Concomitantly, the FeS cluster of the cryo-EM structures are further apart from the Q_o_ site. The FeS of the yeast cyt *bc*_1_ complex is located at the closest distance to the Q_o_ site, as it is constrained by a hydrogen bond from its own ligand His181^RIP1^ to stigmatellin (Figure 2). In contrast, the FeS clusters of the mammalian supercomplex I/III_2_ and the *Candida albicans* complex III are more distant from the Q_o_ site. The distances between ubiquinone and the FeS histidine ligand in these two complexes are larger than 4.5 Å, which is too long for a hydrogen bond. These two positions in the cryo-EM structures likely represent the states of ubiquinone, the product of ubiquinol-oxidation, exiting the catalytic Q_o_ site position.

**Figure 4.**
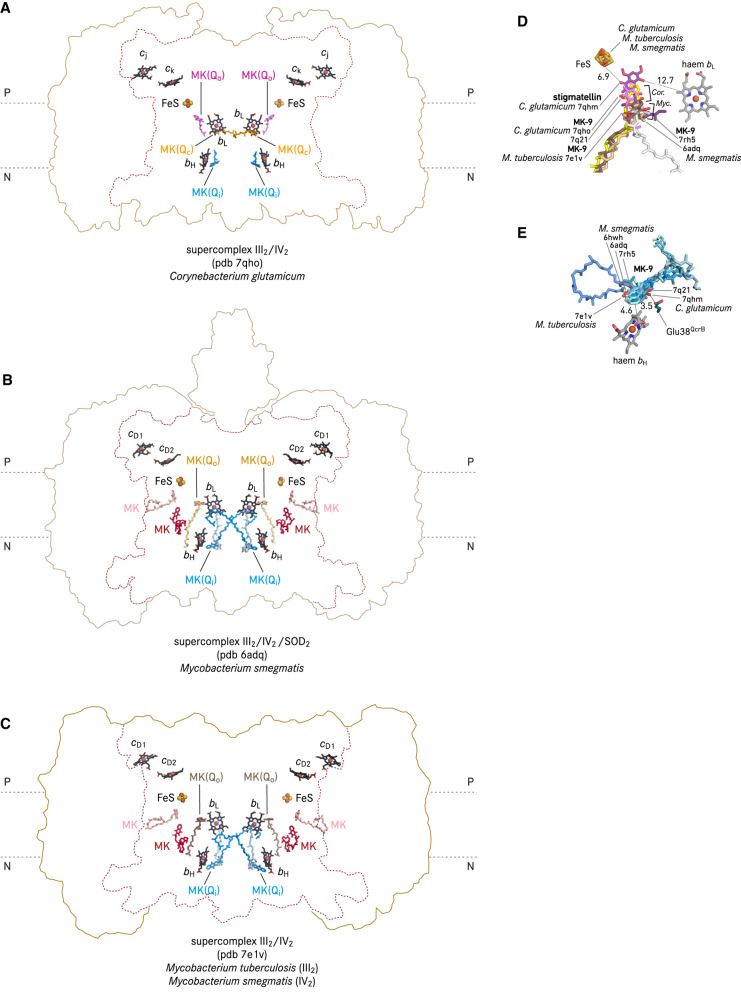
Positions of natural quinone molecules resolved in cyt *bcc*-*aa*_3_ supercomplex. (**A**) cryo-EM structure of cyt *bcc-aa*_3_ supercomplex (supercomplex III_2_/IV_2_) from *Corynebacterium glutamicum* [19]. (**B**) cryo-EM structure of cyt *bcc-aa*_3_ supercomplex (supercomplex III_2_/IV_2_/SOD_2_) from *Mycobacterium smegmatis* [52]. (**C**) cryo-EM structure of a hybrid cyt *bcc-aa*_3_ supercomplex (supercomplex III_2_/IV_2_) with complex III_2_ from *M. tuberculosis* and complex IV_2_ from *M. smegmatis* [65]. (**D**) Comparison of stigmatellin and menaquinone-9 (MK-9) binding positions by superimpositioning the co-ordinates of cyt *bcc-aa*_3_ supercomplex. The QcrB of the *C. glutamicum* cyt *bcc-aa*_3_ supercomplex (pdb 7qhm, with stigmatellin) was used as the reference, and the QcrB of pdb 7qho (*C. glutamicum*), pdb 7q21 (*C. glutamicum*), pdb 6adq (*M. smegmatis*), pdb 7rh5 (*M. smegmatis*), and pdb 7e1v (*M. tuberculosis*) were superposed using secondary structure matching in Coot [112]. The FeS of all structures are shown, whereas only the haem *b*_L_ of pdb 7q21is displayed for the sake of clarity. *Cor*. and *Myc*, respectively, indicate the consensus position of the naphthoquinone ring of MK-9 in the corynebacterial and mycobacterial structures. (**E**) Comparison of MK-9 resolved in the Q_i_ site. The Glu38^QcrB^ side chain and haem *b*_H_ are from pdb 7qhm. All distances are in Å.

In prokaryotes, a co-isolated menaquinone at the Q_o_ site was resolved in two cryo-EM structure of *bcc-aa*_3_ supercomplex from the actinobacterium *Corynebacterium glutamicum* [19,54] (Figure 4A). This menaquinone molecule is positioned in ∼6 Å distance to the closest possible H-bonding partners His355^QcrA^ and Tyr153^QcrB^, respectively (QcrA and QcrB are homologous to mitochondrial Rieske ISP and cyt *b*), and is 9.4 and 13.7 Å apart from FeS and haem *b*_L_, respectively [19]. In a cryo-EM structure of the actinobacterial cyt *bcc-aa*_3_ supercomplex from *Mycobacterium smegmatis*, a menaquinone molecule was described in 14 Å and 16 Å distance from FeS and haem *b*_L_, respectively (Figure 4B) [52]. This binding position agrees with a menaquinone molecule resolved in another *M. smegmatis* cryo-EM structure [64], as well as a menaquinone molecule identified in the hybrid supercomplex composed of the *M. tuberculosis* cyt *bcc* complex and *M. smegmatis* cyt *aa*_3_ oxidase [65] (Figure 4C). By superimposition of the structures of the corynebacterial supercomplex with stigmatellin [19], with menaquinone [19,54], and the mycobacterial supercomplex structures with menaquinone [52,64,65], genus-specific consensus menaquinone binding positions can be deduced (Figure 4D). The locations of FeS in these structures are static. The menaquinone molecules in the two structures of the corynebacterial supercomplex both partially overlap with the stigmatellin binding position, whereas the menaquinone molecules of the three structures of the mycobacterial supercomplex were consistently located closer to the entrance of the quinone exchange cavity (Figure 4D). These experimentally resolved menaquinone molecules likely illustrate a migration path to the catalytic position of menaquinol, which is represented by the transition state analogue stigmatellin [66,67]. Interestingly, the Q_o_ site menaquinone position assigned in a *M. smegmatis* supercomplex (pdb 6hwh, Figure 3D) [53] does not agree with the Q_o_ site menaquinone positions shown in other four actinobacterial supercomplex structures and its naphthoquinone ring was resolved in 21 Å and 19 Å to FeS and the haem *b*_L_ iron [53], therefore this model is not included in Figure 4D.

**Figure 5.**
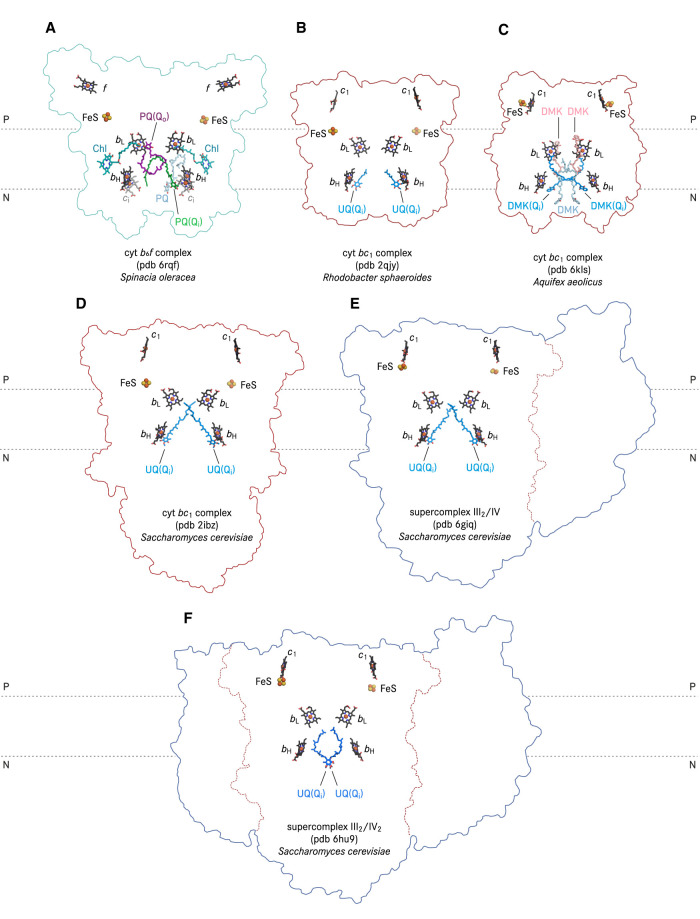
Positions of natural quinone molecules resolved in cyt *b*_6_*f and bc*_1_ complexes. (**A**) cryo-EM structure of cyt *b*_6_*f* complex (pdb 6rqf) from spinach [51]. (**B**) X-ray structure of cyt *bc*_1_ complex (pdb 2qjy) from *Rhodobacter sphaeroides* [69]; (**C**) cryo-EM structure of cyt *bc*_1_ complex (pdb 6kls) from *Aquifex aeolicus* [72]; (**D**) X-ray structure of cyt *bc*_1_ complex (pdb 2ibz) from baker's yeast [67]. The deposited structure contains only one protomer which belongs to the crystallographic asymmetric unit. Here the dimeric structure shown was generated by applying symmetry operation; (**E**) cryo-EM structure of a supercomplex containing a dimeric cyt *bc*_1_ complex and a monomeric cyt *c* oxidase (supercomplex III_2_/IV, pdb 6giq) from baker's yeast [47]; (**F**) cryo-EM structure of a supercomplex containing a dimeric cyt *bc*_1_ complex and a dimeric cyt *c* oxidase (supercomplex III_2_/IV_2_, pdb 6hu9) from baker's yeast [46].

In addition to ubiquinone and menaquinone at the Q_o_ site, a plastoquinone was described in the cryo-EM structure of cyt *b*_6_*f* complex from spinach chloroplasts [51], with its benzoquinone ring 26.4 Å apart from FeS and 16.2 Å from haem *b*_L_. It was described as in an approaching position to the Q_o_ site (Figure 5A). Moreover, the entrance of the Q_o_ site in this structure is partially blocked by the phytyl tail of chlorophyll (Chl), which was suggested to gate the Q_o_ site access [51].

Although quinone molecules were resolved in the Q_o_ site of cyt *bc* complexes in several positions, structural information of the natural substrate in the catalytic relevant position in the Q_o_ site with close distance to electron and proton acceptors is still lacking. So far, only the structures with inhibitors bound at the Q_o_ site suggest the potential proton acceptors for quinol oxidation. Taken together, the cryo-EM structure of the ovine supercomplex I/III_2_ provided a first hint of a co-isolated quinone in the Q_o_ pocket in the context of substrate exchange between complexes I and III. The diverse binding positions of native co-purified ubiquinone, menaquinone and plastoquinone molecules resolved in structures of cyt *bc* complexes, most likely exemplify snapshots of their migration paths in and out of the active site and stand-by positions.

## Quinone binding positions at the Q_i_ site

In contrast with the Q_o_ site characterisation, many X-ray and cryo-EM structures of cyt *bc* complexes described co-purified quinone molecules in the Q_i_ site. A plausible explanation is that the Q_i_ site substrate has to be stabilised within the cyt *b* pocket to ensure a full Q cycle turnover with the two-step reduction to semiquinone and quinol, which is strictly coupled to the oxidation of two quinol molecules in the Q_o_ site (Figure 2). Binding poses of Q_i_ site ubiquinone including ordered water molecules were obtained with high resolution X-ray structures of bovine [38], chicken [39,68], yeast cyt *bc*_1_ complexes (Figure 5D) [17] and that from *Rhodobacter sphaeroides* (Figure 5B) [69]. In brief, the Q_i_ site ubiquinone is consistently located within ca. 5 Å distance to the porphyrin ring of haem *b*_H_ (Figure 3D) in the different structures. Two proposed proton transfer pathways were assigned from the protein surface on the mitochondrial matrix side (the electro-negative side) to Asp229^COB^ and His202^COB^ (yeast numbering, Figs. 2, 3D). Each residue is connected via hydrogen bonds to a carbonyl group of the Q_i_ site ubiquinone. The exact hydrogen bond pattern, whether it is a direct interaction or mediated by water molecules, varies in X-ray structures of the complex from different species [21]. That the binding of the Q_i_ site inhibitor antimycin A replaced the natively occupied ubiquinone with Asp229^COB^ as its direct interaction partner (in the bovine structure, pdb 1ppj) [38].

X-ray crystallographic analysis resolved highly ordered quinone molecules in the Q_i_ site of crystallised cyt *bc*_1_ complex. The power of cryo-EM to better cope with global or local protein dynamics brought forward a higher variety of quinone binding modes at the Q_i_ site. In cryo-EM structures of mitochondrial respiratory chain supercomplexes, of the yeast supercomplex III_2_/IV [47] (Figure 5E) and the ovine supercomplex I/III_2_ [48] (Figure 3A) one ubiquinone molecule was resolved in each Q_i_ site, in a position consistent to the known binding poses in X-ray structures of mitochondrial cyt *bc*_1_ complexes (Figs. 3D, 5D). In contrast, in the cryo-EM structure of yeast supercomplex III_2_/IV_2_ [46], an ubiquinone ring was modelled on the internal two-fold symmetry axis of the dimeric cyt *bc*_1_ complex with two alternate conformations (Figure 5F). The distance from the quinone ring to haem *b*_H_ of each protomer is 15.3 Å.

In actinobacterial respiratory supercomplexes, a menaquinone molecule was identified in the Q_i_ site of the cyt *bcc*-*aa*_3_ supercomplex of *C. glutamicum*, *M. smegmatis*, and *M. tuberculosis* (Figs. 3E, 4A–C) [19,52–54,65]. The interaction mode between haem *b*_H_ and menaquinone is very similar to that of ubiquinone in cyt *bc*_1_ complexes (Figure 3D). In contrast with mitochondrial cyt *bc*_1_ complexes, in which protons could be delivered to the Q_i_ site ubiquinone via a histidine and an aspartate residue, of which the side chains have direct or water-mediated hydrogen bonds to both carbonyl groups of the quinone, the menaquinone molecule resolved in the Q_i_ site of the cyt *bcc* complex from *C. glutamicum* is single hydrogen-bonded directly to a glutamate side chain (Figure 4E) [19]. Interestingly, a second menaquinone was identified near the Q_i_ site of the *bcc* complex from *M. smegmatis* [52], with its naphthoquinone ring in 3.6 Å distance to that of the other menaquinone in the Q_i_ site (Figure 4B). This short distance between the two menaquinone molecules in and close to the Q_i_ site would allow a consecutive reduction from one to the other. Menaquinone and ubiquinone are quinone species of low (−78 mV) and high (+90 mV) redox midpoint potential, respectively [13,14,55–57]. The hyperthermophilic *Aquifex aeolicus* uses demethylmenaquinone (DMK) which has a potential of +36 mV [70], giving it a transitional position in the evolution of cyt *bc* complexes from low to high potentials [71]. The cryo-EM structure of the *A. aeolicus* cyt *bc*_1_ complex with bound DMK molecules at the Q_i_ site (Figure 5C) revealed a 6.1 Å distance from the naphthoquinone ring to haem *b*_H_ [72], which is in good agreement with the binding mode of the Q_i_ site ubiquinone in yeast and *Rhodobacter* homologues as well as the Q_i_ site menaquinone of the actinobacterial cyt *bcc*-*aa*_3_ supercomplex.

The most unique Q_i_ site architecture of cyt *bc* complexes is found in cyt *b*_6_*f* complexes. The position equivalent to the aforementioned ubiquinone and menaquinone ring plane in the Q_i_ site is replaced by a high spin *c*-type haem (haem *c*_i_), which is attached via a single thioether bond to cyt *b*_6_ and which has no amino acid axial ligand [9,10]. A recent cryo-EM structure of spinach cyt *b*_6_*f* complex revealed the position of a plastoquinone molecule at the Q_i_ site (Figure 5A) [51]. The benzoquinone ring of this plastoquinone molecule is 4.4 Å apart from the haem *c*_i_ porphyrin ring. In addition, one of its carbonyl groups is hydrogen-bonded to a propionate carboxylate of haem *c*_i_ in 3.2 Å. Notably, the Q_i_ site plastoquinone breaks the internal two-fold symmetry of cyt *b*_6_*f* complex (Figure 5A). The isoprenoid tail of the Q_i_ site plastoquinone extends into the entrance of the unoccupied Q_i_ site of the other protomer while a second plastoquinone was modelled in a diagonal position with respect to the Q_i_ site plastoquinone, in a position approaching the Q_o_ site of the other protomer [51]. In addition, the Q_i_ site occupancy of plastoquinone seems to be correlated to the orientation of the propionate group of haem *c*_i_, which may control access to a potential proton transfer pathway from the stromal side (the electronegative side) via Asp20 and Arg207 [51]. It was therefore hypothesised that both Q_i_ sites are not simultaneously functional [51].

Whereas high-resolution X-ray structures revealed detailed binding modes of the Q_i_ site ubiquinone in mitochondrial cyt *bc*_1_ complexes, cryo-EM structures more recently provided additional information of ubiquinone positions in the context of supercomplexes, and previously unavailable structures of plastoquinone and menaquinone-occupied Q_i_ sites which show considerably different architecture as compared with mitochondria cyt *bc*_1_ complexes. We anticipate that alternate reaction mechanisms will be required to accomplish quinone reduction and protonation at the Q_i_ site in these complexes.

## Inhibitors bound at Q_o_ or Q_i_ site of cyt *bc* complexes

The use of Q_o_ and Q_i_ site inhibitors was instrumental in studies of cyt *bc*_1_ complexes in order to explore the molecular basis of the Q cycle mechanism and to elucidate electron transfer pathways [58]. Their binding positions in Q_o_ and Q_i_ site, in particular that of stigmatellin [16,17], myxothiazol, UHDBT, NQNO and antimycin A [37] were all analyzed as early as the first X-ray structures of cyt *bc*_1_ complexes were determined (Table 2). Stigmatellin is a semiquinone analogue, i.e. it mimics a transition state of quinol oxidation and reduction [66,67], which is difficult to be captured in protein crystals or cryo-EM specimens with natural substrates. Therefore, its binding poses in the Q_o_ site of cyt *bc*_1_ complex [17] and cyt *bcc* complex [19] provide insights in the catalytic position from which the protons and electrons are released to their respective acceptors. Parallel to fundamental research, cyt *bc*_1_ complex inhibitors are also of great agricultural and medical importance: Azoxystrobin [37,73] belongs to the strobilurins [74], a group of chemically similar compounds [75] which accounted for 27% of the total fungicide worldwide sales in year 2015 [76]. The Q_o_ site inhibitor Famoxadone is a fungicide for crops [77]. Atovaquone [78] is used in a fixed-dose combination with proguanil as antimalarial drug [79–81], and is also used for treating pneumocystis infection [82]. Note that both, atovaquone and strobilurin inhibitors target the Q_o_ site, however, resistances were identified soon after these compounds were made commercially available [81,83–85]. Consequently, development of cyt *bc*_1_ complex inhibitors targeting the Q_i_ site could provide a chance to bypass this issue [86,87]. Interestingly, in the past 5 years, almost all new antimalarial drug candidates resolved in structures of cyt *bc*_1_ complexes published in the RCSB protein data bank (PDB, www.rcsb.org) are Q_i_ site inhibitors (Table 2). This includes the X-ray structures of cyt *bc*_1_ complex inhibited by the antimalarial 4(1H)-pyridones GSK 932121 and GW844520 [88], MJM170 [89], and a 2-pyrazolyl quinolone WDH2G7 [90]. Although X-ray structures can deliver information on protein–ligand interaction with atomic detail, structure-based drug discovery is often hindered by the amount of protein available, time required for crystallisation trials, and conformational heterogeneity or dynamic properties of proteins. The cryo-EM structures of cyt *bc*_1_ complex with bound compounds SCR0911 and GSK 932121 [91] exemplified the scope of cryo-EM structures to characterise binding of drug candidates to target proteins with dynamic properties. Cryo-EM structures of the *Mycobacterium* cyt *bcc*-*aa*_3_ complex with the tuberculosis drug candidate telacebec (Q203) [92] and with TB47 bound at the Q_o_ site demonstrated this approach for bacterial cyt *bc* complexes and supercomplexes [64,65]. Cryo-EM has the advantage of lower sample consumption for single particle analysis as compared with X-ray crystallography. This is especially important for proteins isolated from scarce sources such as patient tissue [93], or from pathogens which are difficult or dangerous to cultivate [94]. In this respect, cryo-EM also opens new possibilities in obtaining structural information of cyt *bc* complexes to develop novel human medications as well as agrochemicals [95–98].

**Table 2.** Structures of cyt *bc* complexes with bound non-native compounds and their application

Position		Year	Non-native compound	pdb	Res (Å)	Method	Origin	Applications
	Q_i_	1998	Antimycin	3bcc	3.70	X-ray	*Gallus gallus*	Research
Q_o_		1998	Stigmatellin	3h1j	3.00	X-ray	*Gallus gallus*	Research
Q_o_		2000	Stigmatellin	1ezv	2.30	X-ray	*Saccharomyces cerevisiae*	Research
Q_o_		2003	Famoxadone	1l0l	2.35	X-ray	*Bos taurus*	Fungicide
Q_o_	Q_i_	2003	NQNO	1nu1	3.20	X-ray	*Bos taurus*	Research
Q_o_		2003	Tridecylstigmatellin	1vf5	3.00	X-ray	*Mastigocladus laminosus*	Research
Q_o_		2003	Tridecylstigmatellin	1q90	3.10	X-ray	*Clamydomonas reinhardtii*	Research
Q_o_		2004	Azoxystrobin	1sqb	2.69	X-ray	*Bos taurus*	Fungicide
Q_o_		2004	HHDBT	1p84	2.50	X-ray	*Saccharomyces cerevisiae*	Research
Q_o_		2004	MOAS	1sqq	3.00	X-ray	*Bos taurus*	Fungicide
Q_o_		2004	Myxothizol	1sqp	2.70	X-ray	*Bos taurus*	Research
Q_o_		2004	UHDBT	1sqv	2.85	X-ray	*Bos taurus*	Research
	Q_i_	2005	Antimycin A	1ppj	2.10	X-ray	*Bos taurus*	Research
Q_o_		2005	Stigmatellin	1pp9	2.10	X-ray	*Bos taurus*	Research
Q_o_		2006	JG144	2fyu	2.26	X-ray	*Bos taurus*	Fungicide
Q_o_		2006	Stigmatellin	2fyn	3.20	X-ray	*Rhodobacter sphaeroides*	Research
Q_o_		2007	NQNO	2e75	3.55	X-ray	*Mastigocladus laminosus*	Research
Q_o_		2008	Crocacin-D iodinated analogue	3cwb	3.51	X-ray	*Gallus gallus*	Fungicide
Q_o_		2008	Stigmatellin	2qjy	2.40	X-ray	*Rhodobacter sphaeroides*	Research
Q_o_	Q_i_	2010	Ascochlorin	3h1l	3.21	X-ray	*Gallus gallus*	Anti-Trypanosomiasis
Q_o_		2010	Azoxystrobin	3l71	2.84	X-ray	*Gallus gallus*	Fungicide
Q_o_		2010	Famoxadone	3l74	2.76	X-ray	*Gallus gallus*	Fungicide
Q_o_		2010	Fenamidone	3l75	2.79	X-ray	*Gallus gallus*	Fungicide
Q_o_		2010	Kresoxim-methyl	3l72	3.06	X-ray	*Gallus gallus*	Fungicide
Q_o_		2010	Kresoxim-methyl iodinated derivative	3h1k	3.48	X-ray	*Gallus gallus*	Fungicide
Q_o_		2010	Triazolone	3l73	3.04	X-ray	*Gallus gallus*	Fungicide
Q_o_		2010	Trifloxystrobin	3l70	2.75	X-ray	*Gallus gallus*	Fungicide
Q_o_		2011	Stigmatellin	2yiu	2.70	X-ray	*Paracoccus denitrificans*	Research
Q_o_		2012	MOA-like (WF3)	3tgu	2.70	X-ray	*Gallus gallus*	Fungicide
Q_o_		2014	Atovaquone	4pd4	3.04	X-ray	*Saccharomyces cerevisiae*	Antimalarial
	Q_i_	2015	4(1H)-pyridone GSK932121	4d6u	4.09	X-ray	*Bos taurus*	Antimalarial
	Q_i_	2015	4(1H)-pyridone GW844520	4d6t	3.57	X-ray	*Bos taurus*	Antimalarial
Q_o_		2015	Famoxadone	5kkz	2.97	X-ray	*Rhodobacter sphaeroides*	Fungicide
Q_o_		2015	MOA-like (Y52)	4u3f	3.23	X-ray	*Gallus gallus*	Fungicide
Q_o_		2016	Fenamidone	5klv	2.65	X-ray	*Bos taurus*	Fungicide
	Q_i_	2016	MJM170	5mni	3.50	X-ray	*Bos taurus*	Anti-Apicomplexan
	Q_i_	2018	2-pyrazolyl quinolone WDH2G7	6haw	3.45	X-ray	*Bos taurus*	Antimalarial
	Q_i_	2018	4(1H)-pyridone GSK932121	6fo0	4.10	cryo-EM	*Bos taurus*	Antimalarial
	Q_i_	2018	SCR0911	5okd	3.10	X-ray	*Bos taurus*	Antimalarial
	Q_i_	2018	SCR0911	6fo6	4.10	cryo-EM	*Bos taurus*	Antimalarial
Q_o_		2019	Azoxystrobin	6nhh	3.00	X-ray	*Rhodobacter sphaeroides*	Fungicide
	Q_i_	2020	Antimycin A	6klv	3.20	cryo-EM	*Aquifex aeolicus*	Research
Q_o_		2021	Telacebec (Q203)	7rh7	3.00	cryo-EM	*Mycobacterium smegmatis*	Anti-Tuberculosis
Q_o_		2021	Telacebec (Q203)	7e1w	2.67	cryo-EM	*Mycobacterium tuberculosis/smegmatis*	Anti-Tuberculosis
Q_o_		2021	TB47	7e1x	2.93	cryo-EM	*Mycobacterium tuberculosis/smegmatis*	Anti-Tuberculosis
Q_o_		2021	Inz-5	7rje	3.30	cryo-EM	*Candida albicans*	Fungicide
Q_o_		2022	Stigmatellin	7qhm	2.80	cryo-EM	*Corynebacterium glutamicum*	Research

This list is not exhaustive, only the first structure of a unique species containing a particular compound, or the structure resolved with the highest resolution are included. The pdb code, resolution (Res) and experimental method of each entry are sourced from RCSB PDB (https://www.rcsb.org).

## Detergent, lipids and the native membrane

Owing to the nature that membrane proteins are located in the lipidic compartments of the cell [99], structural biology studies of membrane proteins have greatly benefited from the use of detergents to solubilise them from their native environment into aqueous solution. Detergent molecules bind to hydrophobic surfaces of membrane proteins and increase their solubility in aqueous environment. Detergents differ in their chemical and physical properties and the selection of the type of detergent is key to prepare well-diffracting membrane protein crystals [100] as well as cryo-EM grids with good contrast and particle distributions [101]. However, detergents compete with the binding of lipids and lipidic compounds such as quinone thus delipidation is unavoidable. Severe delipidation compromises the stability and eventually the integrity of isolated membrane proteins, which may cause artificial structural disorder and may account for poor resolution of X-ray and cryo-EM structures. Reintroducing the detergent solubilised membrane protein back into lipidic cubic phase (L.C.P.) for crystallisation [102,103] and the application of lipidic nanodiscs in solubilisation or reconstitution of isolated membrane protein complexes for cryo-EM specimen preparation [104,105] have shown superior stabilisation effect so as to improve resolution. This can be exemplified by the X-ray structure of *Thermus thermophillus* cyt *caa*_3_ oxidase (2.36 Å resolution, L.C.P. [106]), cryo-EM structures of *Escherichia coli* cyt *bd* oxidase (2.68 Å resolution, nanodiscs [107]) and the cryo-EM structure of *Paracoccus denitrificans* cyt *c* oxidase (2.37 Å resolution, nanodiscs [108]; all resolution of cryo-EM data refer to the FSC = 0.143 criteria for the same basis of comparison). Respiratory chain complexes and supercomplexes in nanodiscs may provide additional information about partitioning of co-purified quinone molecules and their trajectories to fully reflect the native electron transport chain in the hydrophobic environment. Finally, structural studies using *in situ* cryogenic electron tomography (cryo-ET) permits the determination of higher order assemblies of protein complexes as well as structural dynamics directly in cells [109]. Although many technical limitations, such as to resolve small molecules with sufficient resolution still need to be overcome, the rapid and intensive development of cryo-ET [110] will eventually allow to visualise the respiratory chain and photosynthesis complexes in cellular context and maybe in action.

## Perspectives

- Structural biology research of cyt *bc* complexes will contribute to an in-depth understanding of redox-driven proton translocation via the Q cycle and its regulation as well as support the design of fungicides, anti-malarial and anti-tuberculosis drugs.
- Structural characterisation of cyt *bc* complexes from a wide spectrum of species as well as in different types of supercomplexes is important to expand the knowledge on conserved and species-specific binding modes of native substrates, drugs, and inhibitors at the quinone binding sites.
- Structural information on the enzyme-substrate complex and defined catalytic states of cyt *bc* complexes is still lacking. We encourage that the cryo-EM specimens or crystals should be prepared in lipid environment.

## Abbreviations

DMK: demethylmenaquinone
ETC: electron transport chain
ISP: iron-sulfur protein
